# Cohort Profile: Stratifying Resilience and Depression Longitudinally (STRADL): a questionnaire follow-up of Generation Scotland: Scottish Family Health Study (GS:SFHS)

**DOI:** 10.1093/ije/dyx115

**Published:** 2017-07-18

**Authors:** L B Navrady, M K Wolters, D J MacIntyre, T-K Clarke, A I Campbell, A D Murray, K L Evans, J Seckl, C Haley, K Milburn, J M Wardlaw, D J Porteous, I J Deary, A M McIntosh

**Affiliations:** 1 Division of Psychiatry, University of Edinburgh, Edinburgh, UK; 2 School of Informatics, University of Edinburgh, Edinburgh, UK; 3 Centre for Clinical Brain Sciences, University of Edinburgh, Edinburgh, UK; 4 Centre for Genomic and Experimental Medicine, Institute of Genetics and Molecular Medicine, University of Edinburgh, Edinburgh, UK; 5 Generation Scotland, Centre for Genetics and Experimental Medicine, Institute of Genetics and Molecular Medicine, University of Edinburgh, Edinburgh, UK; 6 Aberdeen Biomedical Imaging Centre, University of Aberdeen, Aberdeen, UK; 7 Centre for Cognitive Ageing and Cognitive Epidemiology, University of Edinburgh, Edinburgh, UK; 8 Endocrinology Unit, Centre for Cardiovascular Science, Queen's Medical Research Institute, Edinburgh, UK; 9 MRC Human Genetics Unit, Institute of Genetics and Molecular Medicine, University of Edinburgh, Edinburgh, UK; 10 Health Informatics Centre, University of Dundee, Dundee, UK; 11 Brain Research Imaging Centre, School of Clinical Sciences, University of Edinburgh, Edinburgh, UK; 12 Department of Psychology, University of Edinburgh, Edinburgh, UK

## Why was the cohort set up?

Common health conditions such as heart disease, stroke and depression are a common cause of chronic suffering and economic burden worldwide.^1^ Scotland has a high prevalence of these conditions, and because of its comparatively stable population,^2^ it provides a useful citizenry to study their prevalence and impact. Generation Scotland (GS) is a multi-institutional, cross-disciplinary collaboration aiming to promote research into genetics and health care throughout Scotland.^3^ Between 2006 and 2011, GS undertook its first major study–Generation Scotland: Scottish Family Health Study^1^^,^^4^ (GS:SFHS). This large, family-based intensively phenotyped and genotyped population cohort was designed to examine a diverse range of illnesses such as those aforementioned. The work of GS is especially important to epidemiological research as it provides a means of separating genetic and shared environmental contributions to common non-communicable diseases. Furthermore, the ability to re-contact GS participants and obtain broad consent for future use of their data and samples is especially valuable for prospective studies associated with health outcomes.

In 2015, a strategic award by the Wellcome Trust provided funding for ‘STRADL: Stratifying Resilience and Depression Longitudinally’. This project aimed to re-contact participants from GS:SFHS for a further assessment of mental health, specifically depression. Major depressive disorder (MDD) is a leading cause of global disease burden, with a lifetime prevalence of approximately 10%.^5^^,^^6^. In coming decades, the prevalence and impact of depression will likely increase,^4^ making the understanding of its aetiology of substantial importance to public health. STRADL was designed to investigate the aetiology of depression and its stratification, as it is hypothesized that the diagnosis may group together several causally distinct but symptomatically related syndromes.^7^ The increased kinship among STRADL participants created a rich dataset to conduct genetic studies of MDD aetiology, in addition to examining the complex genetic and environmental interactions which may increase risk for different depression phenotypes.

STRADL was also designed to investigate psychological resilience^8–10^-the ability to ‘escape’ psychopathology despite exposure to known risk factors. Whereas the investigation of resilience may be expected to reveal similar results to studying MDD itself, it has the potential to elucidate protective factors in MDD, even in at-risk individuals. Indeed, examining the variability in response to known MDD risks may not just further our understanding of MDD; a better understanding of resilience mechanisms may also inform future interventions long before the development of the illness.^11^ Ultimately, the work of STRADL has the potential to identify causal mechanisms and pathways of depression sub-types and elucidate mechanisms which give rise to better than expected adjustment.

## Who is in the cohort?

The original GS:SFHS protocol and cohort characteristics have been described extensively elsewhere.^1^^,^^4^ Between 2006 and 2011, 24 084 participants were extensively phenotyped in addition to providing DNA samples for whole-genome genotyping. GS:SFHS was largely female (59%), and generally healthier and wealthier than the Scottish population.

**Figure 1:**
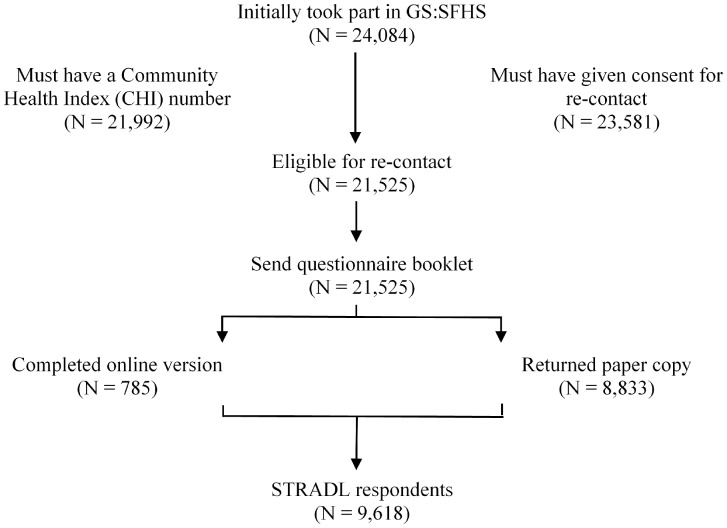
STRADL recruitment flow diagram.

STRADL sought to recruit GS:SFHS participants for a follow-up assessment of mental health and resilience (*N* = 24 084). Individuals were eligible to participate if they had taken part in GS:SFHS, had a Community Heath Index (CHI) number, were alive and living in Scotland, and had given consent for re-contact. A total of 21 525 (89%) individuals from GS:SFHS were eligible for re-contact (Figure 1).

Study packages were sent to potential participants (*N* = 21 525), which consisted of a study invitation letter, detailed information sheet and a paper booklet containing the STRADL questionnaires. Study packages were mailed by an independent party. Participants were given the option to complete the questionnaires on paper and return them via Freepost envelope, or use a given URL link for online submission. The opportunity to return an e-mail address for further contact was also provided.

‘Broad’ consent was obtained from participants, permitting the use of their data for ‘future medical research into health, illness and medical treatment’ without further specification. Participants were informed their data would remain anonymous and would be added to those already held by GS.

A total of 9618 participants responded (45%) at follow-up. A total of 2460 families (*N* = 7158 individuals) of between 2 and 18 family members and 2460 unrelated individuals formed the STRADL cohort. The majority completed the paper version of the questionnaire (*N* = 8833), and 8% completed online (*N* = 785). Henceforth, individuals who participated in STRADL will be referred to as ‘respondents’, and those who did not reply will be referred to as ‘non-respondents’. All components of STRADL received formal, national ethical approval from the NHS Tayside committee on research ethics (reference 14/SS/0039).

Age and sex information were collected in the STRADL, and all other demographic data were obtained from GS:SFHS (Table 1). The STRADL cohort was predominately female (62%) and were older [mean = 50.48 years, standard deviation (SD) = 13.41] than non-respondents [mean = 44.28, SD = 15.70, *t*(21457) = −31.25, *P* < 0.001, Cohen’s *d* = 0.42) at baseline. STRADL respondents were from less socioeconomically deprived areas compared with non-respondents (*t*(19812) = −15.15, *P* < 0.001, Cohen’s *d* = 0.21) in the Scottish Index of Multiple Deprivation (SIMD) 2009.^12^^,^^13^ The STRADL cohort was generally healthier and wealthier, with a different age-sex profile in comparison with GS:SFHS, although important similarities were apparent (Table 1). Although STRADL may not be truly representative of the Scottish population, the sample includes data on participants from all socioeconomic status strata.

**Table 1:** Some baseline comparisons between STRADL respondents and non-respondents and full baseline sample (GS:SFHS)

	Respondents (*N* = 9618)	Non-respondents (*N* = 11907)	GS:SFHS total (*N* = 21525)
Median age (years)			
Male	54	43	48
Female	52	45	48
Gender (% female)	62	57	59
Employment (those aged up to 75 years) (%)			
Unemployed	4	5	5
Retired	18	13	15
Employed (full- or part-time, or self-employed)	71	71	71
Education (%)			
Degree	37	28	32
No qualification	7	9	8
Annual income > £30 000 (%)	63	57	60
SIMD	4123 (1777)	3733 (1875)	3910 (1842)

Abbreviations: STRADL, Stratifying Resilience and Depression Longitudinally; GS, Generation Scotland; GS:SFHS, Generation Scotland: the Scottish Family Health Study; SIMD, the Scottish Index of Multiple Deprivation 2009. With the exception of age and SIMD, values represent percentage; SIMD represents mean rank (SD).

## What has been measured?

A summary of all data collected and their completeness is shown in Table 2. All data were anonymized using a barcode system which linked with a unique participant identification number at the time of data analysis. Paper questionnaires were scanned into an electronic database with detailed in-built validity checks.

**Table 2:** Summary of phenotype data available, and percentage providing valid/useable data (*N* = 9618)

		Phenotype	%
1.		**Demographics**	
	a.	Age	100
	b.	Sex	100
	c.	Email address	79.99
2.		**Medical History**	
	a.	Stoke or mini stroke (TIA)	98.55
	b.	Heart attack or angina	98.44
	c.	Other heart disease	98.16
	d.	Pains in leg muscles	98.07
	e.	Diabetes (blood sugar problems)	98.54
	f.	High blood pressure	98.47
	g.	High blood cholesterol	98.03
3.		**Brief Resilience Scale (BRS)**	98.00
4.		**List of Threatening Experiences (LTE)**	99.00
5.		**Composite International Diagnostic Interview – Short Form (CIDI-SF)**	97.08
6.		**General Health Questionnaire-28 (GHQ-28)**	93.94
	a.	GHQ somatic	98.09
	b.	GHQ anxiety	98.52
	c.	GHQ social dysfunction	98.53
	d.	GHQ depression	95.96
7.		**Alcohol and tobacco consumption**	
	a.	Have you ever smoked tobacco?	99.12
	b.	Are you a current smoker?	46.20
	c.	If yes, how many cigarettes do you smoke in an average week?	–
	d.	If yes, how many cigars do you smoke in an average week?	–
	e.	If yes, how many 25g packets of tobacco do you smoke in an average week?	–
	f.	Do you currently drink any alcoholic drinks?	98.22
	g.	Have you ever felt you should cut down on your drinking?	80.06
	h.	Have people annoyed you by criticising your dinking?	80.13
	i.	Have you ever felt bad or guilty about your drinking?	80.06
	j.	Have you ever had a drink first thing in the morning to steady your nerves or to get rid of a hangover?	80.14
	k.	In an average week, how many units of alcohol do you consume?	79.67
8.		**Coping Inventory for Stressful Situations (CISS)**	
	a.	Task-oriented coping	93.37
	b.	Emotion-oriented coping	94.93
	c.	Avoidance-oriented coping	94.30
	d.	Distraction	95.47
	e.	Social Diversion	96.22

Although data collection was largely cross-sectional, repeated measures of GS:SFHS measures were collected which enabled longitudinal examination. Pearson’s chi-square (χ^2^) tests were conducted to illustrate group differences for categorical data during GS:SFHS. As an alternative to the independent *t* test, comparisons between respondents and non-respondents at baseline are reported using the Mann–Whitney–Wilcoxon (U) test. Wilcoxon signed rank (W) tests have been reported for differences between respondents across GS:SFHS and STRADL as a nonparametric test equivalent to the dependent *t*test. Calculations for group differences and changes over time have ignored the relatedness of the sample, which will be appropriately controlled for in future publication of the data. These differences are shown in Table 3.

**Table 3:** Repeated measures between respondents and non-respondents during GS:SFHS (baselines) and STRADL (follow-up)

	GS:SFHS measures		STRADL measures
	STRADL Non-respondents (*N* = 11907)	STRADL Respondents (*N* = 9618)	Respondents (*N* = 9618)
Currently smoke (%)	21	13*	17**
Currently drink (%)	90	91*	80**
Alcohol units per week	10.3 (12.3)	9.7 (10.8)*	13.9 (22.4)**
Meets SCID criteria for MDD (%)	13	13	–
Meets CIDI-SF criteria for MDD (%)	–	–	16
Meets CIDI-SF criteria for bipolar disorder (%)	–	–	1.3
Meets CIDI-SF criteria for hypomanic episode (%)	–	–	0.4
Total GHQ score	16.4 (9.1)	15.4 (8.4)*	16.9 (9.3)**
GHQ depression score	0.97 (2.4)	0.80 (2.2)*	1.0 (2.2)**
GHQ anxiety score	4.0 (3.8)	3.6 (3.5)*	4.4 (4.2)**

Abbreviations: GS, Generation Scotland; GS:SFHS, Generation Scotland: the Scottish Family Health Study; STRADL, Stratifying Resilience and Depression Longitudinally; SCID, Structured Clinical Interview for DSM-IV; MDD, Major Depressive Disorder; CIDI-SF, Composite International Diagnostic Interview – Short Form; GHQ, General Health Questionnaire. Unless denoted by (%), results represent mean (SD). GHQ scores calculated using the Likert method.

*Significantly different from non-respondents in Wave1 at *P* < 0.05; **significantly different from respondents in Wave 2 at *P* < 0.05.

### Substance use

At baseline 17% of all participants smoked, although respondents were less likely to smoke (13%) compared with non-respondents (21%) [χ^2^(1) = 260.58, *P* < 0.001, *r* = 0.11]. At follow-up, the percentage of respondents smoking increased to 17% [χ^2^(1) = 1563.60, *P* < 0.001, *r* = 0.60].

At baseline 90% of participants drank alcohol, with respondents more likely to drink alcohol than non-respondents [χ^2^(1) = 5.23, *P* = 0.02, *r* = 0.02]. Respondents consumed less units per week (mean = 9.64, SD = 10.79) than did non-respondents (mean = 10.32, SD = 12.25) at baseline [*t*(13221) = 3.39, *P* < 0.001, Cohen’s *d* = 0.06], although this difference was small in magnitude. The number of respondents who drank alcohol decreased at follow-up [χ^2^(1) = 2575.7, *P* < 0.001, *r* = 0.53]. Respondent alcohol consumption substantially increased at follow-up (W = 3149600*, P* < 0.001), although extreme values were reported (mean = 13.91, SD = 22.42, range = 0−914). Respondent’s alcohol consumption was moderately correlated between time points (*r* = 0.50).

### Mental health assessment

GS:SFHS participants were screened for a lifetime history of major depressive disorder (MDD) using the Structured Clinical Interview for DSM-IV Disorders (SCID).^14^ Although the SCID has the potential to make inferences on an array of Axis I disorders, only case/control classifications for MDD were ascertained. The threshold for lifetime prevalence of MDD follows *Diagnostic and Statistical Manual of Mental Disorders*^15^ (DSM) criteria. Where symptoms of either depressed mood or anhedonia have been endorsed, a minimum of four further symptoms must also be endorsed and their clinical significance confirmed (i.e. symptoms lasting nearly all day, every day for a minimum period of 2 weeks).

STRADL participants completed the Composite International Diagnostic Interview–Short Form (CIDI-SF).^16^ The CIDI-SF is a self-report questionnaire measure of psychiatric symptoms, developed from the larger CIDI by the World Health Organization^17^ according DSM-IV^15^ criteria. The CIDI-SF uses a stem-branch logic in which two symptomatic screening questions (symptoms of depressive mood or anhedonia) must be endorsed and reach clinical significance (lasting nearly all day, every day for 2 weeks or more). A minimum of four other symptoms must also be endorsed in addition to at least one screening question. Respondents who meet criteria for lifetime history of MDD reliably meet full diagnostic criteria with excellent accuracy if given the long version of CIDI.^16^ As the CIDI-SF can be completed in a relatively short period (approximately 10 min), it is a scaleable and acceptable measure for epidemiological studies.

At baseline, an identical proportion of respondents (13%) and non-respondents (13%) met criteria for a lifetime history of MDD as established using the SCID, [χ^2^(1) = 0.55, *P* = 0.457, *r* = 0.01]. In STRADL, 16% of respondents met the CIDI-SF criteria for lifetime MDD (*N* = 1506), of whom 16% reported being currently depressed. Lifetime history of MDD was moderately correlated between the two measures (*r* = 0.30).

The General Health Questionnaire-28^18^ (GHQ-28) was administered across time points as a tool used to identify milder psychiatric problems in the general population.^19^ As psychological distress represents a cluster of emotional symptoms linked to depression, the GHQ-28 was used alongside clinical measures to make better distinctions between syndrome and sub-threshold symptoms.^20^^,^^21^ Responses were scored using the Likert method (0‐1‐2‐3) whereby higher scores represent higher levels of psychological distress. Individual domain scores gave information on somatic symptoms, anxiety, social dysfunction and depression.

Non-respondents experienced more psychological distress at baseline than respondents (U = 44888000*, P* < 0.001) although levels of psychological distress increased over time in STRADL respondents (W = 9713600*, P* < 0.001). Total GHQ score of respondents across time points was moderately correlated (*r* = 0.46).

Symptoms of GHQ depression appeared greater in non-respondents than respondents at baseline, (U = 44607000*, P* < 0.001). GHQ depression scores increased in respondents between time points (W = 1409700*, P* < 0.001). GHQ depression scores were moderately correlated (*r* = 0.46) in respondents between assessments.

Symptoms of GHQ anxiety were higher in non-respondents than respondents at baseline, (U = 45042000*, P* < 0.001). Anxiety scores increased in respondents between time points (W = 8937100*, P* < 0.001), and were moderately correlated (*r* = 0.45).

### New measures

Questionnaire measures of psychological resilience,^22^ coping style,^23^ threatening life experiences^24^ and medical conditions were obtained in STRADL, and are summarized in Tables 4, 5 and 6.

**Table 4:** Respondent results of resilience, coping style and psychological distress testing in STRADL

	*n*	Theoretical maximum score	Mean	SD	Median	Range
BRS	9411	5	2.99	0.36	3	1–5
CISS						
Task-oriented	8980	80	54.33	12.28	56	16–80
Emotion-oriented	9130	80	37.61	12.57	37	16–80
Avoidant-oriented	9070	80	39.41	10.52	40	16–80
Distraction-oriented	9182	40	17.46	6.01	17	8–40
Social Diversion-oriented	9254	25	14.33	4.84	15	5–25
GHQ-28						
GHQ-a^a^	9432	21	4.58	3.76	3	0–21
GHQ-b^a^	9476	21	4.37	4.17	3	0–21
GHQ-c^a^	9477	21	7.60	2.48	7	0–21
GHQ-d^a^	9229	21	1.01	2.24	0	0–15
GHQ total	9035	84	16.88	9.28	14	0–65

GHQ scores calculated using the Likert method.

Abbreviations: STRADL, Stratifying Resilience and Depression Longitudinally; SD, Standard deviation; BRS, Brief Resilience Scale; CISS, Coping Inventory for Stressful Situations; GHQ-28, General Health Questionnaire-28.

a GHQ-28 domain scores give information on: (a) somatic symptoms; (b) anxiety/insomnia; (c) social dysfunction; and (d) depression.

**Table 5:** Results from respondents completing the List of Threatening Experiences summarizing the number of individuals who endorsed each event and their ratings of its impact

	*n^1^*	*n^2^*	Theoretical maximum score	Mean	SD	Median	Range
Serious injury or assault to yourself	794	783	3	2.18	0.73	2	1–3
Serious injury or assault to a close relative	1880	1831	3	2.19	0.73	2	1–3
Did a parent, spouse, child or sibling die?	1145	1126	3	2.40	0.71	3	1–3
Close family friend or other relative died	1841	1798	3	1.75	0.74	2	1–3
Separation due to marital difficulties or break of a steady relationship	427	425	3	2.27	0.73	2	1–3
Serious problem(s) with close friend, neighbour or relative	1059	1047	3	2.13	0.68	2	1–3
Made redundant or sacked from job	301	292	3	1.82	0.81	2	1–3
Seeking work unsuccessfully for more than 1 month	367	342	3	1.76	0.73	2	1–3
Major financial crisis (such as losing 3 months’ income)	436	413	3	2.11	0.79	2	1–3
Problems with the police involving court appearance	111	108	3	2.15	0.83	2	1–3
Something of value lost or stolen	372	361	3	1.91	0.76	2	1–3
Yourself or partner gave birth	393	353	3	2.71	0.61	3	1–3

*n^1^*, The number of participants who indicated they experienced the event within the past 6 months.

*n^2^*, The number of individuals who subsequently gave criterion contextual threat ratings of the event.

**Table 6:** Self-reported diagnosis of common illnesses among respondents

	*n*	%
Stoke or mini stroke (TIA)	302	3
Heart attack or angina	466	5
Other heart disease	382	4
Pains in leg muscles when walking or in bed at night	1703	18
Diabetes (blood sugar problems)	575	6
High blood pressure	2257	24
High blood cholesterol	1887	20

The Brief Resilience Scale^22^ (BRS) assessed the ability to ‘bounce back’ from stress. Six questions were answered on a five-point scale from ‘Strongly disagree’ to ‘Strongly agree’. A total score was calculated as the mean of the six items, with appropriate reverse scoring of even-numbered questions. The BRS has previously shown good internal consistency and test-retest reliability.^22^

The Coping Inventory for Stressful Situations (CISS)^23^ was a self-report questionnaire measuring three coping style scales: task-oriented, emotion-oriented and avoidance-oriented coping. Two sub-scales of avoidance-oriented coping were also derived: distraction and social diversion. Each item was rated on a five-point scale from (1) ‘Not at all’ to (5) ‘Very much’. The CISS has proven a robust measure of assessing situation-specific coping strategies, with a stable factor structure and high construct validity.^23^^,^^25^

The List of Threatening Experiences (LTE)^24^ was a self-report measure consisting of 12 common and threatening life events that may have occurred in the 6 months preceding completion. For each threatening event endorsed, criterion contextual threat ratings are measured on a scale from 3 (‘Very bad’) to 1 (‘Not too bad’). The LTE has been shown to have excellent test-retest reliability and high sensitivity.^26^

## How often have they been followed up?

STRADL is a mental health questionnaire follow-up of GS:SFHS. Although data collection was cross-sectional, STRADL becomes a longitudinal cohort because of NHS data linkage using Community Health Index (CHI) numbers which are allocated to every individual registered with a GP in Scotland. The ability to link with routinely collected NHS data will allow validation of the self-reported illness recorded in the study, and provide information on clinical endpoints and follow-up. Furthermore, the use of NHS linkage converts this two-phase cohort study into a potentially lifelong study of resilience and depression. Future parts of the STRADL study will include DNA methylation analysis and depression-focused neuroimaging measures of brain structure, function and connectivity.

As with any epidemiological study, a key question is often whether consenting participants are representative of the population from which they are drawn. As indicated in Table 1, STRADL participants appear to be an older, wealthier, largely female subset of GS:SFHS. This is not surprising, as these characteristics are associated with higher response rates to follow-up surveys.^27^^,^^28^ Whereas results from STRADL may under-represent what would be reflected in the general Scottish population, the cohort size results in large amounts of data which represent the full adult spectrum of ages, sex and demography.

Attrition between time points has been described in Figure 1. It is possible that the response to STRADL (45%) is due to a response bias whereby individuals with mental health difficulties are more likely to respond to a mental health questionnaire when the purpose has been clearly communicated. Although the use of paper questionnaires enables many near-complete participant responses, it is possible that the majority of potential participants forgot to complete or return their questionnaire booklets.

It is worth noting the disparity in the proportions of paper and online responses. Baseline differences between paper and online respondents are presented in Table 7. Several reasons have been hypothesized for these differences. The older demographic of STRADL may have oversampled GS:SFHS participants who did not have access to a personal computer with internet access, or those who were not confident or willing to complete an online survey. Furthermore, the URL provided in the written letter was also very long (53 characters) and it is possible that manually typing a long URL in a browser’s address bar may have been intimidating or inconvenient for many respondents, especially if information technology knowledge was limited.

**Table 7:** Some baseline (GS:SFHS) comparisons between STRADL paper and online respondents

	Paper respondents (*N* = 8833)	Online respondents (*N* = 785)
Median age (years)		
Male	55	50
Female	52	43
Gender (% female)	63	46
Employment (those aged up to 75 years) (%)		
Unemployed	4	3
Retired	16	8
Employed (full- or part-time, or self-employed)	73	81
Education (%)		
Degree	36	47
No qualification	7	2
Annual income > £30 000	62	74
SIMD	4115 (1777)	421409 (1781)

Abbreviations: GS:SFHS, Generation Scotland: Scottish Family Health Study; STRADL, Stratifying Resilience and Depression Longitudinally; SIMD, the Scottish Index of Multiple Deprivation 2009. With the exception of age and SIMD, values represent percentage. SIMD represents mean (SD).

## What has it found? Key findings and publications

Baseline differences between GS:SFHS and STRADL are summarized above (Tables 1 and 3), and differences between online and paper respondents are given in Table 7. This cohort represents a new and potentially valuable data resource to examine incident depressive symptoms, longitudinal outcomes and mechanisms of psychological resilience. No articles have yet been published with these data, but the power of this resource is extensive. Genomic and pedigree-based approaches to these data will enable us to estimate trait heritability and the contribution of shared and non-shared environmental effects to depression.^29^ These data may provide clues as to how people can modify behaviour to reduce their risk of depression and psychological distress. Furthermore, STRADL will allow us to conduct genetic epidemiological analysis on indices of mental health, building upon existing data held by GS.

## What are the main strengths and weaknesses?

The STRADL cohort includes important phenotypes to allow population-based genetic and epidemiological research on the stratification of MDD and resilience. The strengths of this cohort lie in the repeated assessment of mood disorders, psychological distress and substance use, making it a valuable dataset to investigate the pathogenic mechanisms that underlie psychopathology, in addition to making longitudinal predictions on depression and resilience. As data can be linked anonymously to NHS records, STRADL can be converted from a cross-sectional analysis into a longitudinal cohort covering a wide range of clinically relevant outcomes. Furthermore, the availability for longitudinal sampling is of benefit in obtaining repeated measures of mental health and resilience that might be missed by a single measure.^30^^,^^31^

Further, specific limitations of this cohort warrant consideration. First, like other population cohorts such as UK Biobank,^32^^,^^33^ STRADL participants were more likely to be graduates and to come from less socioeconomically disadvantaged areas. Nevertheless, participants from all socioeconomic strata were represented in both baseline (GS:SFHS) and follow-up samples.

Differences in prevalence rates of MDD were found in STRADL (using the CIDI-SF), compared with the use of the SCID at baseline. This may be because the SCID is administered face-to-face by trained researchers, and may have better psychometric properties than the self-reported CIDI-SF.^34^ However, previous research suggests that the diagnostic classifications obtained using the CIDI-SF accurately reflect those made in the larger Composite International Diagnostic Interview.^16^ In future, the CIDI-SF will be compared with the SCID and with linked NHS records so that a comparison of each technique can be made and potential issues of recall bias can be overcome.

Overall, the GS:SFHS follow-up (STRADL) represents a valuable resource to investigate the stratification of depression and mechanisms of psychological resilience in a large, family-based, cohort.

## Can I get hold of the data? Where can I find out more?

Non-identifiable information from this cohort will be made available to researchers throughout the UK and to international collaborators, and are available from the GS Access Committee at [resources@generationscotland.org].

Profile in a nutshell

- GS Wave 2 is a re-contact study of GS:SFHS which aimed to collect longitudinal measures of depressive symptoms, major depressive disorder (MDD) and new measures of psychological resilience to identify their aetiology.
- From 2015–2016, 21 525 participants from the GS:SFHS cohort were re-contacted and asked to take part in a further mental health reassessment. In total, 9618 GS:SFHS individuals consented to take part and provided useable re-contact data, with a response rate of 45%.
- The re-contact data included questionnaire measurement of medical conditions, psychological resilience, threatening life events and repeated measures of mental health and substance use. Consent for health record linkage was provided, in addition to whole-genome genotyping.
- This large, family-based cohort represents a valuable resource to investigate the aetiology and stratification of depression, mechanisms of psychological resilience and the familial aggregation of many relevant traits.

## Funding

STRADL is supported by the Wellcome Trust through a Strategic Award (reference 104036/Z/14/Z). The Chief Scientist Office of the Scottish Government Health Department (CZD/16/6) and the Scottish Funding Council (HR03006) provided core support for Generation Scotland. A.M.M. is supported by the Dr Mortimer and Theresa Sackler Foundation. D.J.M. is supported by an NRS Fellowship, funded by the CSO. J.S., J.M.W., K.L.E., D.J.P., I.J.D. and A.M.M. are members of the Centre for Cognitive Ageing and Cognitive Epidemiology which also supports I.J.D.; funding from the Medical Research Council and Biotechnology and Biological Sciences Research Council is gratefully acknowledged (MR/K026992/1).
